# Supplementary material for: Social exclusion concepts, measurement, and a global estimate
Source: PLoS One. 2024 Feb 28;19(2):e0298085. doi: 10.1371/journal.pone.0298085 (PMC10901322; doi:10.1371/journal.pone.0298085)
Supplement: S1 File — (DOCX) [file pone.0298085.s001.docx]

**Supplementary information on methodology**

**A. Group-specific societal poverty headcounts**

The observed overall poverty rate at the SPL can be expressed as the population weighted average of the poverty rates concerning the group at risk of social exclusion, on the one hand, and the rest of the people, on the other:

${povrate\_SPL}_{ct}={povrate\_SPL}_{gct}\times share{pop}_{gct}+{povrate\_SPL}_{(-g)ct}\times\left( 1-share{pop}_{gct} \right).$ (A1)

Dividing by ${povrate\_SPL}_{gct}$both sides of the equation:

$$\frac{{povrate\_SPL}_{ct}}{{povrate\_SPL}_{gct}}=share{pop}_{gct}+\frac{{povrate\_SPL}_{(-g)ct}}{{povrate\_SPL}_{gct}}\times\left( 1-share{pop}_{gct} \right).$$

Defining $k_{gct}=\frac{{povrate\_SPL}_{gct}}{{povrate\_SPL}_{(-g)ct}}$:

$\frac{{povrate\_SPL}_{ct}}{{povrate\_SPL}_{gct}}=share{pop}_{gct}+\frac{1}{k_{gct}}(1-share{pop}_{gct})$. (A2)

And solving for the variable of interest:

${povrate\_SPL}_{gct}=\frac{{povrate\_SPL}_{ct}}{share{pop}_{gct}+\frac{1}{k_{gct}}(1-share{pop}_{gct})}.$ (A3)

From the previous equation we observe ${povrate\_SPL}_{ct}$ and $share{pop}_{gct}$, but no *k_gct_*. We approximate *k_gct_* by the ratio of the available proxy poverty rates: $\hat{k}_{gct}=\frac{{povrate\_proxy}_{gct}}{{povrate\_proxy}_{(-g)ct}}$ to get:

$${povrate\_\hat{SPL}}_{gct}=\frac{{povrate\_SPL}_{ct}}{share{pop}_{gct}+\frac{1}{\hat{k}_{gct}}(1-share{pop}_{gct})} (A4)$$

Our method extrapolates the poverty rate differential at the available poverty line to the SPL. We should use proxy poverty lines as similar to the SPL as possible for two related reasons: one, the composition of the poor may change depending on the poverty line even across a short range of values due to socioeconomic reasons, and two, the composition of the poor necessarily changes as the number of poor in a group reaches the total population of that group. In this regard, we can see in Equation (A1) that for $\hat{k}_{gct}>1$ the estimated group-specific SPL poverty rate (${povrate\_\hat{SPL}}_{gct}$) may be greater than 1 depending on the observed overall SPL poverty rate (${povrate\_SPL}_{ct}$) and the size of group at risk of social exclusion ($share{pop}_{gct}$). Note that $\hat{k}>1$ is the relevant case as the poverty rates are always larger for the groups at risk of social exclusion.

We first select the proxies for the SPL poverty rates according to the criteria shown in Table 1 trying to use poverty rates at those poverty lines closer to the SPL when data for several poverty rates at different poverty lines are available.

The second issue, which is especially apparent when extrapolating poverty rate differentials concerning a small at-risk group from a country with relatively low poverty rates to a country with a high SPL poverty rate. The most notorious example is the estimation of the number of the poor LGBTI in African countries using the poverty rate differentials affecting this group in the UK and the US.

We address this challenge by establishing a linear correcting factor for $\hat{k}_{gct}$ as a function of the ratio $r_{gct}=\frac{{povrate\_SPL}_{ct}}{{povrate\_proxy}_{ct}}$. It is worth pointing out that ${povrate\_proxy}_{ct}={povrate\_proxy}_{gct}\times share{pop}_{gct}+ {povrate\_proxy}_{(-g)ct}\times(1-share{pop}_{gct})$ is different for each vulnerable group. We assume that $\hat{k}_{gct}$ converges linearly to 1 across the interval $r_{gct}\in[1,2]$ and remains equal to 1 thereafter. The rationale is that, as can be seen in Equation A1, when $\hat{k}_{gct}=1$ the estimated group-specific SPL poverty rate becomes equal to the observed overall SPL poverty rate. More formally, the function is defined as follows:

$\hat{k}_{gct}=\left\{ \begin{matrix} \hat{k}_{gct}^{0} & \text{if} r_{gct}\leq1 \\ 2\hat{k}_{gct}^{0}-1+\left( 1-\hat{k}_{gct}^{0} \right)r_{gct} & \text{ if} 1<r_{gct}<2 \\ 1 & \text{if} r_{gct}\geq2 \end{matrix} \right.$ (A5)

where $\hat{k}_{gct}^{0}=\frac{{povrate\_proxy}_{gct}}{{povrate\_proxy}_{(-g)ct}}$.

Graphically it takes the following form:

$$1$$

$$\hat{k}_{gct}$$

$$\hat{k}_{gct}^{0}$$

$$2$$

$$1$$

$$r_{gct}$$

This correcting factor is arbitrary although it seems reasonable and conservative. Thus, we establish that once the overall SPL poverty rate doubles the overall proxy rate the group at risk of social exclusion has the same poverty incidence as the general population. For example, our upper-bound estimate for the share of the adult population belonging to the LGBTI community in Angola in 2017 is based on an estimation from (51) for the US, which is 4.4%. The estimated differential incidence of poverty between LGBTI and non-LGBTI individuals is derived from available data in the UK (71) and the US (72), with the estimated poverty rate among the LGBTI population being 15.6%, and the estimated poverty rate among the non-LGBTI population being 11.3%. Thus $\hat{k}^{0}=\frac{15.6}{11.3}=1.4$.

Existing estimates show an overall poverty rate of 11.4 percent. On the other hand, the estimated overall poverty rate at the SPL in Angola in 2017 was 48.98 percent (40). Therefore, in this case: $r=\frac{48.98}{11.4}=4.3>2$. Then, based on expression (A5): $\hat{k}=1$, and it follows from equation (A4) that the estimated LGBTI population’s poverty rate at the SPL in Angola in 2017 is the same as among the general population: 48.98%. The adjustment in $\hat{k}$, that is, in the estimated poverty rate differential between a given vulnerable group and the rest of the population is smaller in other cases. For example, as regards the LGBTI community in the US in 2017, we have already seen that the estimated share of the US adult population belonging to the LGBTI community was 4.4% (51).

According to Badgett et al. (72)’s data, the estimated poverty rate among the adult LGBTI people at the national poverty line in 2017 was 21.6% whereas the estimated poverty rate among the non-LGBTI people was 15.7%, thus $\hat{k}^{0}=\frac{21.6}{15.7}=1.4$. Preceding estimates imply an overall poverty rate of 16%. On the other hand, the estimated overall poverty rate at the SPL in the US in 2017 is 19.25% (40). Therefore, in this case $r=\frac{19.25}{16}=1.2$. Then, based on expression (A5): $\hat{k}=2\times\frac{21.6}{15.7}-1+(1-\frac{21.6}{15.7})\times\frac{19.25}{16}=1.3$. Finally, it follows from equation (A4) that the estimated LGBTI population’s poverty rate at the SPL in the US in 2017 is $\left( \frac{0.1925}{0.044+\frac{1}{1.3}\left( 1-0.044 \right)} \right)\times100=24.7\%$.

For each vulnerable group, Table S1.1 shows the number of countries that bind the restriction $r_{gct}\leq1$.

**Table S1.1** Number of countries in the sample by $r_{gct}$ interval and vulnerable group

|  | $r_{gct}\leq1$ | $1<r_{gct}<2$ | $r_{gct}\geq2$ | Total N |
| --- | --- | --- | --- | --- |
| Children | 38 | 113 | 44 | 195 |
| Women | 75 | 54 | 66 | 195 |
| People with disability | 120 | 64 | 11 | 195 |
| LGBTI people | 19 | 53 | 123 | 195 |
| Indigenous people | 21 | 23 | 15 | 59 |
| Afrodescendants | 5 | 28 | 5 | 38 |
| Religious minorities | 59 | 101 | 35 | 195 |

**B. Correcting for overlapping at-risk populations based on socioeconomic status**

To account for the total number of people at risk of being socially excluded based on socioeconomic status adding up all at-risk groups’ poverty numbers we sequentially estimate the intersection between each group and all the preceding ones in the sequence and subtract the resulting intersection from the corresponding unadjusted total. We use the *men* category as a residual group that comprises those individuals that are not included in the preceding groups.

First, once we have accounted for societal poor children, we should discount them from the remaining at-risk groups that include children (Intersecting group CH in Table 2). Regarding the age composition of the poverty headcount of at-risk group *g* (${povhc\_\hat{SPL}}_{gct}$), in general we assume the same age distribution as among the poor in general, thus we estimate the number of children among the societal poor in at-risk group *g*, for *g* including women, indigenous peoples, Afrodescendants, and religious minorities, in country *c* and year *t* as:

${povhc\_\hat{SPL}}_{(CH)gct}={povhc\_\hat{SPL}}_{gct}\times{sharehc\_\hat{SPL}}_{(CH)ct},$ (A6)

where ${sharehc\_\hat{SPL}}_{(CH)ct}$ is easily computed on the basis of the estimates for the children group in step 2 as:

${sharehc\_\hat{SPL}}_{(CH)ct}=\frac{{povhc\_\hat{SPL}}_{(CH)ct}}{{povhc\_SPL}_{ct}}.$ (A7)

To illustrate, on the basis of the procedure describe above in S1, Section A, we estimate that 16.6 million children were living below the SPL in the US in 2017. Given that we estimate that there were a total of $62.6$ million people living below the SPL in the US in 2017, it is straight forward to calculate using equation (A7) that a $\frac{16.6}{62.6}\times100=26.5\%$ of them were children. In addition, since there were 34 million women living below the SPL in the US in 2017, we can estimate based on equation (A6) that among this figure, $34\times0.265=9$ million were (female) children.

Only in the case of the PWD poverty headcount do we assume the same age distribution as among the PWD group as a whole (instead of the age distribution among the poor in general), given that the prevalence of disability is highly correlated with age and moreover adults are who determine the poverty status of children in the household. Thus, in this case we have:

${povhc\_\hat{SPL}}_{(CH)(PWD)ct}={povhc\_\hat{SPL}}_{(PWD)ct}\times{sharePWDpop}_{(CH)ct},$ (A8)

where ${sharePWDpop}_{(CH)ct}$ is the share of children within the population of PWD in country *c* and year *t*.

To illustrate, based on available data, we estimate that in 2017, 11.8 percent of people with severe disabilities in Egypt were children. Moreover, based on the procedure presented in Section A above, we estimate that 0.8 million people with severe disabilities were living below the SPL in Egypt in 2017. Accordingly, based on equation (A8), we canestimate that there were $0.8\times0.118=0.1$ million children with severe disabilities living below the SPL in Egypt in that year.

Second, once we have accounted for societal poor adult women, we should discount them from the remaining at-risk groups that include women (Intersecting group FE in Table 2). Regarding the gender composition of the poverty headcount of the at-risk group *g* (${povhc\_\hat{SPL}}_{gct}$), we may estimate the share of women either based on poverty data broken down by gender or assuming the same gender distribution than among the poor in general depending on the available data for each at risk group. Thus, for those groups for which we lack poverty data broken down by gender (persons with disabilities, Afrodescendants in countries other than those from the LAC region, and religious minorities) we assume the same gender distribution than among the poor in general to estimate the number of adult women among the societal poor of the at-risk group *g* in country *c* and year *t* as:

${povhc\_\hat{SPL}}_{(FE)(-CH)gct}={povhc\_\hat{SPL}}_{gct}\times{(1-sharehc\_\hat{SPL}}_{(CH)ct})\times{sharehc\_\hat{SPL}}_{(FE)ct},$ (A9)

Where ${sharehc\_\hat{SPL}}_{(FE)ct}$ is easily computed on the basis of the estimates for the women group in step 2 as:

${sharehc\_\hat{SPL}}_{(FE)ct}=\frac{{povhc\_\hat{SPL}}_{(FE)ct}}{{povhc\_SPL}_{ct}}$. (A10)

To illustrate, based on the procedure described in Section A above, we estimate that 11.7 million women were living below the SPL in Egypt in 2017. Since we estimate that there were $22.8$ million people living below the SPL, it is straight forward to calculate using equation (A10) that a $\frac{11.7}{22.8}\times100=51.3\%$ of people living below the SPL were women. Similarly we estimate that there were 0.8 million people with severe disabilities living below the SPL in the Egypt in 2017. Based on this information, we can estimate based on equation (A9) that among this population there were $0.8\times(1-0.118)\times0.513=0.36$ million adult women.

For those groups for which we have poverty data broken down by gender (LGBTI, indigenous people, and Afrodescendants in Latin American and the Caribbean countries) we estimate the number of adult women among the societal poor of the corresponding at-risk group *g* in country *c* and year *t* as:

${povhc\_\hat{SPL}}_{(FE)(-CH)gct}={povhc\_\hat{SPL}}_{gct}\times{(1-sharehc\_\hat{SPL}}_{(CH)ct})\times{shareghc\_proxy}_{(FE)ct},$ (A11)

Where ${shareghc\_proxy}_{(FE)ct}$, the share of women among the poor of the corresponding at-risk group at the available proxy poverty line, can be directly taken from the shelf, as in the case of LGBTI, or, as in the case of IP and Afrodescendants in LAC countries, estimated as:

${shareghc\_proxy}_{(FE)ct}=\frac{{povhc\_proxy}_{(FE)gct}}{{povhc\_proxy}_{gct}}$, (A12)

where, using data already used in step 2, we have that:

${povhc\_proxy}_{\left( FE \right)gct}={pop}_{(FE)gct}\times{povrate\_proxy}_{(FE)gct},$ (A13)

and

${povhc\_proxy}_{gct}={pop}_{(FE)gct}\times{povrate\_proxy}_{(FE)gct}+{pop}_{(MA)gct}\times{povrate\_proxy}_{(MA)gct}$. (A14)

For example, regarding Afrodescendants in Brazil in 2017, we have the following estimates: Afrodescendant population was approximately 105.4 million people (57); percentage of women in the population: 50.81% (46); Afrodescendant population living below the SPL: 36.4 million (40, 73 and S1, Section A procedure); percentage of children among the population living below the SPL: 34.45% (40, 1 and S1, Section A procedure); ECLAC’s (non-extreme) poverty rates among Afrodescendant women: 20.5%, and among Afrodescendant men: 20.1% (73). Assuming the same share of women among Afrodescendants as among the general population and based on equations A12-A14 we may estimate that the percentage of women among the Afrodescendants living in poverty in Brazil in 2017 was:

$\left( \frac{105.4\times0.5081\times0.205}{105.4\times0.5081\times0.205+105.4\times\left( 1-0.5081 \right)\times0.201} \right)\times100=51.3\%$.

Finally, based on the preceding estimates and on equation (A11) we may estimate that there were 36.4\times\left(1-0.3445\right)\times0.513=12.2 million Afrodescendant adult women living below the SPL in Brazil in 2017.

Third, once we have accounted for societal poor adult men with disabilities, we should discount them from the remaining at-risk groups that include disabled men (intersecting group PWD in Table 2). For this purpose, we assume that PWD represent the same share of the remaining at-risk groups’ poor adult men than among the poor adult men in general. Thus, we have:

$povh{c\_\hat{SPL}}_{\left( MA \right)\left( -CH \right)\left( PWD \right)gct}=povh{c\_\hat{SPL}}_{gct}\times{(1-sharehc\_\hat{SPL}}_{(CH)ct})\times shareh{c\_\hat{SPL}}_{\left( MA \right)ct}\times{share(MA)(-CH)hc\_\hat{SPL}}_{(PWD)ct},$ (A15)

where ${share(MA)(-CH)hc\_\hat{SPL}}_{(PWD)ct}=\frac{{povhc\_\hat{spl}}_{(MA)(-CH)(PWD)ct}}{{povhc\_\hat{spl}}_{(MA)(-CH)ct}}.$

For example, based on the UK’s Office for National Statistics (58), Fontenot et al. (68) and Jolliffe and Prydz (40)’s data and S1 A procedure we estimate that there were 0.5 million Afrodescendant people living below the SPL in the UK in 2017. Moreover, following previous steps we estimate that the percentage of children among the SPL poor was 23.51%; the percentage of men among the SPL poor was 47.73%; the number of male adults living below the SPL was 3.8 million; and the number of severely disabled male adults living below the SPL was 0.2 million. Based on these estimates and equation (A15) we may estimate that there were $0.5\times\left( 1-0.2351 \right)\times0.4773\times\frac{0.2}{3.8}=0.01$ million severely disabled afrodescendant male adults living below the SPL in the UK in 2017.

Fourth, once we have accounted for societal poor adult LGBTI men without disabilities, we should discount them from the remaining at-risk groups that include LGBTI men (Intersecting group LGBTI in Table 2). For this purpose, we assume that LGBTI people represent the same share of the remaining at-risk groups’ poor adult men without disabilities than among the poor adult men in general. Thus, we have:

$povh{c\_\hat{SPL}}_{\left( MA \right)\left( -CH \right)\left( -PWD \right)(LGBTI)gct}=povh{c\_\hat{SPL}}_{gct}\times{(1-sharehc\_\hat{SPL}}_{(CH)ct})\times shareh{c\_\hat{SPL}}_{\left( MA \right)ct}\times(1-{share(MA)(-CH)hc\_\hat{SPL}}_{(PWD)ct})\times{share(MA)(-CH)hc\_\hat{SPL}}_{(LGBTI)ct},$ (A16)

where ${share(MA)(-CH)hc\_\hat{SPL}}_{(LGBTI)ct}=\frac{{povhc\_\hat{spl}}_{(MA)(-CH)(LGBTI)ct}}{{povhc\_\hat{spl}}_{(MA)(-CH)ct}}.$

For example, based on the Pew Research Center (78), Panagariya and Mukim (74) and Jolliffe and Prydz (40)’s data and S1 A procedure we estimate that there were 99.5 million people belonging to a religious minority living below the SPL in India in 2017. Moreover, following previous steps we estimate that the percentage of children among the SPL poor was 33.11%; the percentage of men among the SPL poor was 51.98%; the percentage of severely disabled people among male adults living below the SPL was 5.27%; the number of male adults living below the SPL was 136.3 million; and the number of LGBTI male adults living below the SPL was 2.5 million. Based on these estimates and equation (A16) we may estimate that there were $99.5\times\left( 1-0.3311 \right)\times0.5198\times(1-0.0527)\times\frac{2.5}{136.3}=0.6$ million LGBTI non-severely disabled male adults belonging to a religious minority living below the SPL in the India in 2017.

Fifth, once we have accounted for societal poor indigenous adult men, we should discount them from the remaining at-risk groups that include indigenous men (Intersecting group IP in Table 2). In fact, the only remaining at risk group that intersects with the group IP is the *religious minorities* group (the group *men* is a residual category), whose data is disaggregated in several religious affiliations, one of which, *Folk Religions*, may be used to approximate the intersection between the groups IP and REMI. Thus, we take from the shelf the population of indigenous peoples that belongs to a religious minority in country *c* in year *t*: ${pop}_{(IP)(REMI)ct}.$ For example, based on Pew Research Center (78)’s data we estimate that there were 0.2 million people belonging to a folk religion in Australia in 2020 who we directly assume to be indigenous people belonging to a religious minority (note that only the 0.9% of the population is affiliated to a folk religion).

Assuming a constant share of this intersection across age and gender groups we can easily compute the adult male population in this subgroup: ${pop}_{(MA)\left( -CH \right)(IP)(REMI)ct}$. The percentage of male adults in the population was 38.19% in Australia in 2017 (46). Assuming the same demographic composition among the indigenous people belonging to a religious minority we estimate that there were $0.2\times0.3819=0.08$ million indigenous male adults belonging to a religious minority.

Finally, assuming the same poverty incidence within this subgroup as among the group *religious minorities* in general, we get the headcount of interest as follows:

${povhc\_\hat{SPL}}_{(MA)\left( -CH \right)(IP)(REMI)ct}={pop}_{(MA)\left( -CH \right)(IP)(REMI)ct}\times\left( \frac{povh{c\_\hat{SPL}}_{\left( MA \right)\left( -CH \right)\left( REMI \right)ct}}{{pop}_{\left( MA \right)\left( -CH \right)\left( REMI \right)ct}} \right).$ (A17)

Concluding the Australian example, following previous steps, we estimate that there were $0.95$ million male adults belonging to a religious minority in Australia in 2017, of which $0.2$ million were living below the SPL. Based on these estimates and equation (A17) we estimate that there were $0.08\times\frac{0.2}{0.95}=0.02$ million indigenous male adults belonging to a religious minority living below the SPL.

Finally, regarding the residual group *men*, we determine the total number of non-double-counted societal poor men as the residual or difference between the total number of societal poor people in country *c* and year *t* and the total number already accounted for as members of the previous at-risk groups:

${povhc\_\hat{SPL}\_nointersected}_{(MA)ct}={povhc\_SPL}_{ct}-\sum_{g} ({povhc\_\hat{SPL}}_{gct}- {povhc\_\hat{SPL}\_intersected}_{gct}),$ (A18)

Where ${povhc\_SPL}_{ct}={povrate\_SPL}_{ct}\times{pop}_{ct}$ and ${povhc\_\hat{SPL}\_intersected}_{gct}$ is the sum of the different intersections between the societal poor of group *g* and those from the preceding groups in the sequence as estimated previously and shown in Table 2. For instance, for the case of the indigenous people we have that:

${povhc\_\hat{SPL}\_intersected}_{(IP)ct}={povhc\_\hat{SPL}}_{\left( CH \right)(IP)ct}+{povhc\_\hat{SPL}}_{(FE)\left( -CH \right)(IP)ct}+{povhc\_\hat{SPL}}_{(MA)\left( -CH \right)(PWD)(IP)ct}+{povhc\_\hat{SPL}}_{(MA)\left( -CH \right)(-PWD)(LGBTI)(IP)ct}$. (A19)

Regarding indigenous people in the US in 2017, for example, note that we conservatively estimate that there were 3.9 million, out of which 0.75 million were living below the SPL. Among these 0.75 million there are many who would have shown up when accounting for previous vulnerable groups: children, women, people with severe disabilities and LGBTI people. In this regard, following previous steps we would have estimated that there were 0.2 million indigenous children, 0.3 million indigenous adult women, 0.01 million indigenous adult men with severe disabilities and 0.01 million indigenous LGBTI adult men without severe disabilities living below the SPL. These partial intersections add up using equation (A19) to a total of $0.2+0.3+0.01+0.01=0.52$ million intersected indigenous people living below the SPL. Subtracting this from the initial total we get the number of non-double counted indigenous people, that is, $0.75-0.52=0.23$ million indigenous straight cisgender adult men without severe disabilities living below the SPL. In equation (A18) the latter is one of the addends that we subtract from the total number of people living below the SPL in the US in 2017 to get the total number of non-intersected people living below the SPL, that is, the number of people living below the SPL who are not children, women, severely disabled, LGBTI, indigenous, Afrodescendant, or member of a religious minority.

It is now straightforward to compute the sum of the intersections between the group of societal poor men and those from the previous at-risk groups negatively as the difference between the total number of societal poor men and the amount that do not belong to any of the previous at- risk groups:

${povhc\_\hat{SPL}\_intersected}_{(MA)ct}={povhc\_\hat{SPL}}_{(MA)ct}-{povhc\_\hat{SPL}\_nointersected}_{(MA)ct}$. (A20)

For example, based on World Bank (46 and 67) and Jolliffe and Prydz (40)’s data and Annex 1A procedure we estimate that there were 38.3 million men living below the SPL in Indonesia in 2017, of which there are, according to previous steps, 16.1 million that do not intersect with any previous vulnerable group, thus that are not double counted. Equivalently, using equation (A20) we calculate that there are $38.3-16.1=22.2$ million intersected men living below the SPL in Indonesia in 2017, that is, that are included at least in one of the previous vulnerable groups.

**C. Correcting for overlapping at-risk populations based on socioeconomic status and other circumstances**

Starting from the total country number of people at risk of being socially excluded based on socioeconomic status (step 3), we then add all women victims of gender-based violence minus those already accounted for because of poverty status. We estimate the intersection between poverty and gender-based violence on the basis of data on the incidence of GBV by income level or poverty level. Thus, we first note that:

${GBVrate}_{(FE)act}={GBVrate}_{(FE)a(SPL)ct}\times{povrate\_SPL}_{\left( FE \right)act}+{GBVrate}_{(FE)a(-SPL)ct}(1-{povrate\_SPL}_{\left( FE \right)act}),$ (A21)

Where ${GBVrate}_{(FE)act}$ represents the percentage of women victims of gender based violence in age group *a*, country *c* and year *t*; ${GBVrate}_{(FE)a(SPL)ct}$ is the corresponding incidence rate among women living below the societal poverty line and ${GBVrate}_{(FE)a(-SPL)ct}$ the incidence among those living above that line. ${povrate\_SPL}_{\left( FE \right)act}$ stands for the SPL poverty rate among women in age group *a*, country *c* and year *t*.

Solving for the variable of interest and defining $s_{act}=\frac{{GBVrate}_{(FE)a(SPL)ct}}{{GBVrate}_{(FE)a(-SPL)ct}}$ we have:

${GBVrate}_{(FE)a(SPL)ct}=\frac{{GBVrate}_{(FE)act}}{{povrate\_SPL}_{\left( FE \right)act}+(\frac{1}{s_{act}})\times(1-{povrate\_SPL}_{\left( FE \right)act})}$ (A22)

There are two unknowns in this equation: ${povrate\_SPL}_{\left( FE \right)act}$ and $s_{act}$. Instead of ${povrate\_SPL}_{\left( FE \right)act}$ we use ${povrate\_\hat{SPL}}_{\left( FE \right)act}$ as estimated in Step 2. Regarding $s_{act}$, we use an estimate of this ratio, $\hat{s}_{act}$, based on some auxiliary sources that provide data on the incidence of GBV by income level or poverty status. We note that most of the data available report the incidence of GBV by income quantiles. We use simple interpolations to estimate the incidence corresponding to the SPL percentile. The final expression takes the following form:

$\hat{GBVrate}_{(FE)a(\hat{SPL})ct}=\frac{{GBVrate}_{(FE)act}}{{povrate\_\hat{SPL}}_{\left( FE \right)act}+(\frac{1}{\hat{s}_{act}})\times(1-{povrate\_\hat{SPL}}_{\left( FE \right)act})}.$ (A23)

Note that due to data constraints we assume that ${GBVrate}_{(FE)act}$ and $\hat{s}_{act}$ are constant across age groups. Finally, it is straight forward to estimate the intersection between SPL poverty and gender-based violence as follows:

${povhc\_\hat{SPL}}_{(FE)a(GBV)ct}=\hat{GBVrate}_{(FE)a(\hat{SPL})ct}\times{povhc\_\hat{SPL}}_{(FE)act},$ (A24)

where ${povhc\_\hat{SPL}}_{(FE)act}$ is the SPL poverty headcount of women in age group *a*, country *c* and year *t* as estimated in Step 2. Note that we assume the same age distribution among the poor females as among females in general to estimate the number of poor females with 15 to 17 years of age, on the one hand, and the number of poor females ages 18 to 49, on the other.

We can illustrate this estimation procedure by means of its application to Cambodia’s data in 2017. The estimated percentage of victims of gender based violence in the previous 12 months among women aged 15-49 years in Cambodia in 2014 was 10.9% (65, we impute this estimate to year 2017); the estimated ratio between the percentage of victims of gender based violence among poor women and the percentage of victims among non-poor women in Cambodia in 2000 was $s=1.37$, we impute this estimate to year 2017); following previous steps, the estimated poverty rate at the SPL among adult women was 20.43%, which represents 1.1 million adult women in absolute terms. Based on these data and equation (A23) we estimate that the percentage of victims of gender based violence among SPL poor adult women is $\frac{0.109}{0.2043+\frac{1}{1.37}(1-0.2043)}=13.9\%$. Finally, based on equation (A24) we estimate that there were $1.1\times0.139=0.15$ million adult women living below the SPL and victims of gender based violence in Cambodia in 2017.

We should then add all forcefully displaced persons not accounted already because of poverty status or as victims of GBV. The intersection between forced displacement and SPL poverty (${povhc\_\hat{SPL}}_{(FDP)ct}$) is estimated on the basis of auxiliary data on the incidence of poverty among foreigners as compares with nationals following the same procedure as in Step 2. Finally, assuming that the incidence of gender-based violence is constant across non-poor subgroups, the intersection between non-poor forcibly displaced women and gender based violence is estimated on the basis of some previous results as:

${GBVhc}_{(FE)(-\hat{SPL})(FDP)ct}=\hat{GBVrate}_{(FE)(-\hat{SPL})ct}\times{pop}_{(FE)(-\hat{SPL})(FDP)ct},$ (A25)

Where ${GBVhc}_{(FE)(-\hat{SPL})(FDP)ct}$ is the total country number of non-poor forcibly displaced women that suffer gender based violence in country *c* and year *t*; $\hat{GBVrate}_{(FE)(-\hat{SPL})ct}$ is the estimated incidence of gender based violence among non-poor women; and ${pop}_{(FE)(-\hat{SPL})(FDP)ct}$ is the total country number of non-poor forcibly displaced women.

Note regarding equation (A25) that the total country number of non-poor forcibly displaced women is easily computed subtracting from the total country number of forcibly displaced women (estimated assuming for the forcibly displaced people the same demographic composition as the general population) the total country number of forcibly displaced women living below the SPL (estimated using S1 A and B procedures). Moreover, the share of victims of gender based violence among non-SPL poor women is easily calculated from equation (A21) replacing the rest of components by the corresponding estimate.
