# Supplementary material for: Social exclusion concepts, measurement, and a global estimate
Source: PLoS One. 2024 Feb 28;19(2):e0298085. doi: 10.1371/journal.pone.0298085 (PMC10901322; doi:10.1371/journal.pone.0298085)
Supplement: S1 Table — (DOCX) [file pone.0298085.s002.docx]

**S1 Table.** **Computing the number of socially excluded populations while avoiding double-counting**

| **At-risk group** | **SPL poverty** | | | **Intersection description** | **Intersecting group** | **Assumptions on intersections^1^** |
| --- | --- | --- | --- | --- | --- | --- |
|  | **Total** | **Intersections** |  | |  |  |
| Children, CH | ${povhc\_\hat{SPL}}_{\left( CH \right)ct}$ | -- | -- | | -- | N.a. |
| Women, FE | ${povhc\_\hat{SPL}}_{\left( FE \right)ct}$ | ${povhc\_\hat{SPL}}_{\left( FE \right)(CH)ct}$ | Female children SPL poor | | CH | Age among poor women follows the same distribution as the entire poor population |
| Persons with Disabilities, PWD | ${povhc\_\hat{SPL}}_{\left( PWD \right)ct}$ | ${povhc\_\hat{SPL}}_{\left( CH \right)(PWD)ct}$ | Children with disabilities SPL poor | | CH | Age among poor PWDs follows the same distribution as the entire PWD population |
|  |  | ${povhc\_\hat{SPL}}_{(FE)\left( -CH \right)(PWD)ct}$ | Female adults with disabilities SPL poor | | FE | The gender distribution among poor PWDs is the same as the entire poor population |
| LGBTI people | ${povhc\_\hat{SPL}}_{\left( LGBTI \right)ct}$ | ${povhc\_\hat{SPL}}_{\left( FE \right)(LGBTI)ct}$ | Female LGBTI SPL poor | | FE | n.a. |
|  |  | ${povhc\_\hat{SPL}}_{(MA)(PWD)(LGBTI)ct}$ | Male with disabilities LGBTI SPL poor | | PWD | The incidence of disability among poor LGBTI people is the as the entire poor population |
| Indigenous Peoples, IPs | ${povhc\_\hat{SPL}}_{\left( IP \right)ct}$ | ${povhc\_\hat{SPL}}_{\left( CH \right)(IP)ct}$ | Indigenous children SPL poor | | CH | Age among poor IPs follows the same distribution as the entire poor population |
|  |  | ${povhc\_\hat{SPL}}_{(FE)\left( -CH \right)(IP)ct}$ | Indigenous female adults SPL poor | | FE | n.a. |
|  |  | ${povhc\_\hat{SPL}}_{(MA)\left( -CH \right)(PWD)(IP)ct}$ | Indigenous male adults with disabilities SPL poor | | PWD | The incidence of disability among poor IPs is the same as the entire poor population |
|  |  | ${povhc\_\hat{SPL}}_{(MA)\left( -CH \right)(-PWD)(LGBTI)(IP)ct}$ | Indigenous male adults without disabilities LGBTI SPL poor | | LGBTI | The share of LGBTI people among poor male IPs is the same as among poor men in general |
| Afrodescendants, ADs | ${povhc\_\hat{SPL}}_{\left( AD \right)ct}$ | ${povhc\_\hat{SPL}}_{\left( CH \right)(AD)ct}$ | Afrodescendant children SPL poor | | CH | Age among poor ADs follows the same distribution as the entire poor population |
|  |  | ${povhc\_\hat{SPL}}_{(FE)\left( -CH \right)(AD)ct}$ | Afrodescendant female adults SPL poor | | FE | The gender distribution among poor ADs is the same as among the entire poor population (no assumption for Latin American countries, for which poverty data by ethnicity/race is disaggregated by gender) |
|  |  | ${povhc\_\hat{SPL}}_{(MA)\left( -CH \right)(PWD)(AD)ct}$ | Afrodescendant male adults with disabilities SPL poor | | PWD | The incidence of disability among poor ADs is the same as among the entire poor population |
|  |  | ${povhc\_\hat{SPL}}_{(MA)\left( -CH \right)(-PWD)(LGBTI)(AD)ct}$ | Afrodescendant male adults without disabilities LGBTI SPL poor | | LGBTI | The share of LGBTI people among poor male ADs is the same as among poor men in general |
| Religious minorities, REMI | ${povhc\_\hat{SPL}}_{\left( REMI \right)ct}$ | ${povhc\_\hat{SPL}}_{\left( CH \right)(REMI)ct}$ | Religious minority children SPL poor | | CH | Age among poor people pertaining to religious minorities follows the same distribution as the entire poor population |
|  |  | ${povhc\_\hat{SPL}}_{(FE)\left( -CH \right)(REMI)ct}$ | Religious minority female adults SPL poor | | FE | The gender distribution among poor people pertaining to religious minorities is the same as among the entire poor population |
|  |  | ${povhc\_\hat{SPL}}_{(MA)\left( -CH \right)(IP)(REMI)ct}$ | Religious minority Indigenous male adults SPL poor | | IP | The incidence of poverty among IPs pertaining to religious minorities is the same as among the entire religious minority population |
|  |  | ${povhc\_\hat{SPL}}_{(MA)\left( -CH \right)(PWD)(-IP)(REMI)ct}$ | Religious minority non-Indigenous male adults with disabilities SPL poor | | PWD | The incidence of disability among poor people pertaining to religious minorities is the same as among the entire poor population |
|  |  | ${povhc\_\hat{SPL}}_{(MA)\left( -CH \right)(-PWD)(LGBTI)(-IP)(REMI)ct}$ | Religious minority non-Indigenous male adults without disabilities LGBTI SPL poor | | LGBTI | The share of LGBTI people among poor men pertaining to religious minorities is the same as among poor men in general |

Source: authors.

Notes: Assumptions initially refer to a particular pairwise intersection (e.g., between Indigenous people and female groups). When intersections of a higher order are involved (e.g., between *adult* Indigenous people and females), and no specific information on those intersections is available, we assume that the distribution of the additional category (e.g., females) is constant across the other categories involved in the intersection (e.g., we assume that the share of females among Indigenous people is constant across age groups, i.e., the share of females among *adult* Indigenous people is the same as among the entire Indigenous population). As such, it should be noted that assumptions are cumulative over the sequence of intersections.
