# Supplementary material for: Social exclusion concepts, measurement, and a global estimate
Source: PLoS One. 2024 Feb 28;19(2):e0298085. doi: 10.1371/journal.pone.0298085 (PMC10901322; doi:10.1371/journal.pone.0298085)
Supplement: S2 Table — (DOCX) [file pone.0298085.s003.docx]

**S2 Table.** **Data sources to estimate the number and share of vulnerable populations worldwide, circa 2017**

| **Vulnerable groups** | **Number of observations (countries and territories)** | **Extrapolation of data?** | **Sources** | **Disaggregation details** |
| --- | --- | --- | --- | --- |
| Women | 195 | No | World Bank (2021c) ’s Population Estimates and Projection Database  United Nations (2019)’s World Population Prospects Database | All ages |
| Children | 195 | No | World Bank (2021c) ’s Population Estimates and Projection Database  United Nations (2019)’s World Population Prospects Database | 0-17 years of age |
| People with disabilities | Regional data | Regional | World Health Organization and World Bank (2011) | Severe and moderate categories. Data disaggregated by gender and age |
| LGBTI people | 14 | World | OECD (2019a)  Layte et al. (2006)  Uhrig (2013)  Wilson et al. (2020)  Statistics Canada (2021)  Valfort (2017) | Self-identify as lesbians, gay men or bisexuals (adults)  Self-identified as homosexual or bisexual (18-64, by gender)  Self-identify as lesbians/gay, bisexuals or other (16+)  Self-identify with a minority sexual identity (gay, lesbian, bisexual, and other sexual minority identities), (18+, by gender)  LGBTQ2+ (15+, by gender)  Self-identify LGBTI (18+) (US), LGB (18+, by gender) |
| Indigenous peoples | 59 | No | Stidsen (2007); Sobrevila (2008); OECD (2019b); and Davis-Castro (2020, 2021), who compiles data from several sources, including UNICEF and FUNPROEIB Andes (2009), World Bank’s LAC Equity Lab (2021), ECLAC and Fondo para el Desarrollo de los Pueblos Indígenas de América Latina y el Caribe (FILAC) (2020), World Bank Group (2015), and ECLAC (2014) | All ages |
| Afrodescendants | 44 | No | United Nations Statistics Division (2021)  World Bank (2018); Statistics Canada (2017); UK Office for National Statistics (2013); U.S. Census Bureau (2020); France Ministry for Europe and Foreign Affairs (2019); Istat (2021); INE (2021); CIA (2020); and Seif (2006) | All ages, disaggregated by gender  All ages |
| Religious minorities | 233 | No | Pew Research Center (2015) | Religious minorities include non-majority groups that do not exceed 25 percent of religious affiliation in a country. All ages |
| Victims of gender-based violence | 103 | Regional | World Bank (2021d) based on data of the United Nations Statistics Division (UNSD) | We use the variable Proportion of women subjected to physical and/or sexual violence in the last 12 months (% of women aged 15-49)  We estimate the number of GBV victims who are children (15-17 years old) and those who are adult (18-49 years old) assuming that the incidence is constant across age groups |
| Forcibly displaced populations | 167 | No | United Nations High Commissioner for Refugees (2020) | For each asylum country and year, we compute the total number of FDP as the sum of Refugees under UNHCR's mandate; Asylum-seekers; IDPs of concern to UNHCR; Venezuelans displaced abroad; Stateless persons; and Others of concern over all countries of origin |

Source: Authors
