# Supplementary material for: Social exclusion concepts, measurement, and a global estimate
Source: PLoS One. 2024 Feb 28;19(2):e0298085. doi: 10.1371/journal.pone.0298085 (PMC10901322; doi:10.1371/journal.pone.0298085)
Supplement: S3 Table — (DOCX) [file pone.0298085.s004.docx]

**S3 Table. Studies estimating poverty incidence within vulnerable populations**

| **Source** | **Poverty measure** | | **Sample features** | **Year** | **Number of countries** |
| --- | --- | --- | --- | --- | --- |
| **Women and children** |  | |  |  |  |
| Jolliffe and Prydz (2021) | Social poverty line | | Overall | 1990-2017 | 166 |
| Silwal et al. (2020) | $1.90, $3.20, and $5.50 IPLs | | By age group | 2017 | 149 |
| LIS (2021) | 50% of the Median | | By age group | 1978-2019 | 52 |
| World Bank 2021 PEBs (World Bank 2021e) | $1.90, $3.20, and $5.50 IPLs | | By gender | 2006//2019 | 125 |
| OECD (2019a) | 50% of the Median | | By gender | 2011//2017 | 42 |
| **People with disabilities** | **Relevant concept** | **Poverty measure** | **Sample features** | **Year** | **Number of countries** |
| Fontenot et al. (2018) 1/ | Any disability | US Census Bureau’s multi poverty thresholds measure | Overall  (18-64 years of age) | 2017 | 1 |
| Eurostat (2021a) 2/ | Some or severe activity limitation | At-risk-of-poverty measure | Overall | 2019 | 36 |
| Mitra et al. (2013) | If a person reports a severe or extreme difficulty in, or is unable to do, any of the following: 1) seeing/recognizing people across the road (while wearing glasses or lenses); 2) moving around; 3) concentrating or remembering things; 4) taking care of themself, he or she is identified as having a disability | Alkire and Foster’s (2011) multidimensional poverty measure | Overall  (18–65 years of age) | 2004 | 15 |
| **LGBTI people** | **Relevant concept** | **Poverty measure** | **Sample features** | **Year** | **Number of countries** |
| Uhrig (2013) | Self-identify LGB or other | 50% of Median equalized household income | Overall  (16+ years of age) | 2012 | 1 |
| Badgett et al. (2019) | LGBT | National poverty line | By gender  (18+ years of age) | 2017 | 1 |
| **Ips** | **Poverty measure** | | **Sample features** | **Year** | **Number of countries** |
| ECLAC (2021) 3/ | ECLAC’S poverty and extreme poverty measures | | By gender | 2000-2019 | 11 |
| ILO (2019) | $1.90, $3.20 and $5.50 a day poverty lines | | By gender | 2011//2018 | 7 regions/ groups |
| **Ads** |  | |  |  |  |
| ECLAC (2021) | ECLAC’S poverty and extreme poverty measures | | By gender | 2000-2019 | 11 |
| Fontenot et al. (2018) | US Census Bureau’s multi poverty thresholds measure | | Overall | 2017 | 1 |
| Seif (2006) | Social status | | Overall | 2006 | 1 |
| **Religious minorities** | **Poverty measure** | | **Sample features** | **Year** | **Number of countries** |
| Heath and Yaojun (2015) | 60 per cent of the median income | | Overall  (16+ years of age) | 2011 | 1 |
| Panagariya and Mukim (2014) | Lakdawala Lines | | Overall | 2010 | 1 |
| **GBV** | **Relevant concept** | **Poverty measure** | **Sample features** | **Year** | **Number of countries** |
| Walby and Allen (2004) | Domestic violence or sexual assault (including various grades of severity and attempts) | Estimated income percentile based on SPL poverty rate | Women  (16-59 years of age) | 2001 | UK |
| Altınay and Arat (2009) | Physical violence at least once by their husbands | Estimated income percentile based on SPL poverty rate | Ever married women | 2007 | Turkey |
| Usta et al. (2015) | Physical violence by an intimate partner during past year | Estimated wealth index percentiles based on SPL poverty rates | Ever married women (15-49 years of age) | 2005, 2007 | Egypt, Jordan |
| O’Donnell et al. (2002) | Physical, sexual or psychological violence by an intimate partner | $C15,000 | Ever married women | 1993 | Canada |
| Kishor and Johnson (2004) | Spousal violence in the past 12 months | Estimated wealth percentiles based on SPL poverty rates | Ever married women  (Age 15-49) | 1995//2002 | Cambodia, Dominican Rep., Egypt, Haiti, India, Nicaragua, Zambia |
| Rivera-Rivera et al. (2004) | Low-level acts of emotional and physical violence (e.g., control of activities, not allowing women to have a job, insults, and slaps to the face); More serious physical violence, including having been struck with an object, burned, or locked up; Extremely serious physical or emotional violence, such as strangling attempts and threats with a knife or gun by her most recent partner in the previous year | Estimated socioeconomic level percentile based on SPL poverty rate | Ever partnered women  (ages 15-49)  (Cuernavaca local sample) | 1998 | Mexico |
| Flake (2005) | Ever pushed, shaken, or attacked by partner | Estimated socioeconomic status percentile based on SPL poverty rate | Women currently living with a partner  (Age 15-49) | 2000 | Peru |
| **FDP** | **Relevant concept** | **Poverty measure** | **Sample features** | **Year** | **Number of countries** |
| Eurostat (2021a) | Citizenship: Non-EU28 countries nor reporting country | People at risk of poverty or social exclusion | By gender  (18+ years of age) | 2011-2020 | 37 |
| Eurostat (2021a) | Citizenship of their parents: Foreign country | At-risk-of poverty (AROP) measure | Overall  (Age 0-17) | 2011-2020 | 37 |

Source: Authors

1/ “Following the Office of Management and Budget’s (OMB) Statistical Policy Directive 14, the U.S. Census Bureau uses a set of dollar value thresholds that vary by family size and composition to determine who is in poverty. If a family’s total monetary income is less than the applicable threshold, then that family and every individual in it are considered in poverty. The official poverty thresholds are updated annually for inflation using the Consumer Price Index (CPI-U). The official poverty definition uses money income before taxes or tax credits and excludes capital gains and noncash benefits (such as Supplemental Nutrition Assistance Program benefits and housing assistance). The thresholds do not vary geographically” (Fontenot et al. 2018, p. 47).

2/ *People* *at risk of poverty or social exclusion* “corresponds to the sum of persons who are: at risk of poverty after social transfers, severely materially deprived or living in households with very low work intensity. Persons are counted only once even if they are affected by more than one of these phenomena. Persons are considered to be at risk of poverty after social transfers, if they have an equivalized disposable income below the risk-of-poverty threshold, which is set at 60 % of the national median equivalized disposable income. Severely materially deprived persons have living conditions severely constrained by a lack of resources, they experience at least 4 out of 9 following deprivations items: cannot afford i) to pay rent or utility bills, ii) keep home adequately warm, iii) face unexpected expenses, iv) eat meat, fish or a protein equivalent every second day, v) a week holiday away from home, vi) a car, vii) a washing machine, viii) a color TV, or ix) a telephone. People living in households with very low work intensity are those aged 0-59 living in households where the adults (aged 18-59) work 20% or less of their total work potential during the past year.” (Eurostat 2021b).

3/ The *extreme poverty line* is calculated by ECLAC as the cost of a basic food basket in those countries. “The basic food basket is constructed to satisfy the average energy requirements of the population, using a structure of goods and prices given by consumption patterns observed in a reference group and adjusted for basic dietary balances”. In turn, the *poverty line* “is obtained by multiplying the extreme poverty line by a factor that expresses expenditure on non-food goods and services. Unlike in the case of the basic food basket, for which energy requirements provide exogenous criteria for efficiency evaluation, there are no clear normative parameters for establishing a threshold of non-food goods and services consumption for the poverty line. For that reason, the ratio between total spending and food spending —the Orshansky coefficient— in the reference population is used, without specifying what type of needs are to be met with that amount” (ECLAC 2019, pp. 21-23).
