# Supplementary material for: Social exclusion concepts, measurement, and a global estimate
Source: PLoS One. 2024 Feb 28;19(2):e0298085. doi: 10.1371/journal.pone.0298085 (PMC10901322; doi:10.1371/journal.pone.0298085)
Supplement: S5 Table — (DOCX) [file pone.0298085.s006.docx]

**S5 Table. Populations at risk of social exclusion by dimensions of exclusion in South Asia, 2017**

|  | **Lower bound** | | **Upper bound** | |
| --- | --- | --- | --- | --- |
|  | **N**  **(millions)** | **%**  **Total** | **N**  **(millions)** | **%**  **Total** |
| **Children** |  |  |  |  |
| *Population* | 620.1 | 34.6 | 620.1 | 34.6 |
| Poor | 191.4 | 34.7 | 191.4 | 34.7 |
| *Socially excluded not double counted* | **191.4** |  | **191.4** |  |
|  |  |  |  |  |
| **Women** |  |  |  |  |
| *Population* | 866.9 | 48.4 | 866.9 | 48.4 |
| Poor | 266.8 | 48.3 | 266.8 | 48.3 |
| (Intersection: CH) | (92.6) |  | (92.6) |  |
| *Socially excluded not double counted* | **174.3** |  | **174.3** |  |
|  |  |  |  |  |
| **People with disabilities** |  |  |  |  |
| *Population* | 52.6 | 2.9 | 284.3 | 15.9 |
| Poor | 18.9 | 3.4 | 99.9 | 18.1 |
| (Intersection: CH and FE) | (9.9) |  | (54.1) |  |
| *Socially excluded not double counted* | **8.9** |  | **45.8** |  |
|  |  |  |  |  |
| **LGBTI people** |  |  |  |  |
| *Population* | 29.1 | 1.6 | 51.6 | 2.9 |
| Poor | 9.0 | 1.6 | 15.9 | 2.9 |
| (Intersection: FE and PWD) | (6.0) |  | (12.3) |  |
| *Socially excluded not double counted* | **3.0** |  | **3.7** |  |
|  |  |  |  |  |
| **Indigenous people** |  |  |  |  |
| *Population* | 108.1 | 6.0 | 108.1 | 6.0 |
| Poor | 53.3 | 9.7 | 53.3 | 9.7 |
| (Intersection: CH, FE, PWD & LGBTI) | (36.9) |  | (40.8) |  |
| *Socially excluded not double counted* | **16.5** |  | **12.5** |  |
|  |  |  |  |  |
| **Afrodescendants** |  |  |  |  |
| *Population* | 0.0 | 0.0 | 0.0 | 0.0 |
| Poor | 0.0 | 0.0 | 0.0 | 0.0 |
| (Intersection: CH, FE, PWD & LGBTI) | (0.0) |  | (0.0) |  |
| *Socially excluded not double counted* | **0.0** |  | **0.0** |  |
|  |  |  |  |  |
| **Religious minorities** |  |  |  |  |
| *Population* | 353.0 | 19.7 | 353.0 | 19.7 |
| Poor | 114.6 | 20.8 | 114.6 | 20.8 |
| (Intersection: CH, FE, PWD, LGBTI & IP) | (79.7) |  | (88.6) |  |
| *Socially excluded not double counted* | **34.8** |  | **26.5** |  |
|  |  |  |  |  |
| **Men** |  |  |  |  |
| *Population* | 926.0 | 51.6 | 926.0 | 51.6 |
| Poor | 285.2 | 51.7 | 285.2 | 51.7 |
| (Intersect.: CH, PWD, LGBTI, IP, AD & RELI) | (162.0) |  | (187.4) |  |
| *Socially excluded not double counted* | **123.2** |  | **97.8** |  |
|  |  |  |  |  |
| **Victims of gender based violence** |  |  |  |  |
| *Victims of gender based violence* | 106.8 | 6.0 | 142.2 | 7.9 |
| (Intersection: SPL poor) | (40.0) |  | (53.3) |  |
| *Socially excluded not double counted* | **66.8** |  | **88.9** |  |
|  |  |  |  |  |
| **Forcibly displaced people** |  |  |  |  |
| *Forcibly displaced people* | 6.1 | 0.3 | 6.1 | 0.3 |
| (Intersection: SPL poor & victims GBV) | (2.7) |  | (2.8) |  |
| *Socially excluded not double counted* | **3.3** |  | **3.3** |  |
|  |  |  |  |  |
| **TOTAL** |  |  |  |  |
| *Population* | 1,792.9 | 100.0 | 1792.9 | 100.0 |
| *Socially excluded* | **622.1** | **34.7^a^** | **644.2** | **35.9^a^** |

Source: Authors’ estimates.

Notes: (^a^) % World total population. See section 3 for further details on intersections. The lower bound estimates are based on the following assumptions: (1) victims of GBV are restricted to women aged 15 to 49; (2) the population of PWD is based on the most restrictive definition of disability (severe disability); (3) the population of LGBTI people is based on all available data; (4) the population of IPs is based on the lower estimate available in our sources for each country. On the other hand, the upper bound estimates are based on the following assumptions: (1) the prevalence of GBV is extrapolated to the whole adult female group; (2) the population of PWD is based on the least restrictive definition of disability (at least moderate disability); (3) the size of the LBGTI group in the US (4.4%) is extrapolated to the rest of countries; (4) the population of IPs is based on the larger estimate available in our sources for each country. See section 4 for further details.
